# Pharmacokinetics and Pharmacodynamics of Salvinorin A and *Salvia divinorum*: Clinical and Forensic Aspects

**DOI:** 10.3390/ph14020116

**Published:** 2021-02-03

**Authors:** Andreia Machado Brito-da-Costa, Diana Dias-da-Silva, Nelson G. M. Gomes, Ricardo Jorge Dinis-Oliveira, Áurea Madureira-Carvalho

**Affiliations:** 1 IINFACTS-Institute of Research and Advanced Training in Health Sciences and Technologies, Department of Sciences, University Institute of Health Sciences (IUCS), CESPU, CRL, 4585-116 Gandra, Portugal; a26127@alunos.cespu.pt (A.M.B.-d.-C.); ngomes@ff.up.pt (N.G.M.G.); aurea.carvalho@iucs.cespu.pt (Á.M.-C.); 2 UCIBIO-REQUIMTE, Laboratory of Toxicology, Department of Biological Sciences, Faculty of Pharmacy, University of Porto, 4050-313 Porto, Portugal; 3 LAQV-REQUIMTE, Laboratório de Farmacognosia, Departamento de Química, Faculdade de Farmácia, Universidade do Porto, R. Jorge Viterbo Ferreira, 228, 4050-313 Porto, Portugal; 4 Department of Public Health and Forensic Sciences, and Medical Education, Faculty of Medicine, University of Porto, 4200-319 Porto, Portugal

**Keywords:** hallucinogens, herbal highs, opioid receptors, pharmacodynamics, pharmacokinetics, *Salvia* spp.

## Abstract

*Salvia divinorum* Epling and Játiva is a perennial mint from the Lamiaceae family, endemic to Mexico, predominantly from the state of Oaxaca. Due to its psychoactive properties, *S. divinorum* had been used for centuries by Mazatecans for divinatory, religious, and medicinal purposes. In recent years, its use for recreational purposes, especially among adolescents and young adults, has progressively increased. The main bioactive compound underlying the hallucinogenic effects, salvinorin A, is a non-nitrogenous diterpenoid with high affinity and selectivity for the κ-opioid receptor. The aim of this work is to comprehensively review and discuss the toxicokinetics and toxicodynamics of *S. divinorum* and salvinorin A, highlighting their psychological, physiological, and toxic effects. Potential therapeutic applications and forensic aspects are also covered in this review. The leaves of *S. divinorum* can be chewed, drunk as an infusion, smoked, or vaporised. Absorption of salvinorin A occurs through the oral mucosa or the respiratory tract, being rapidly broken down in the gastrointestinal system to its major inactive metabolite, salvinorin B, when swallowed. Salvinorin A is rapidly distributed, with accumulation in the brain, and quickly eliminated. Its pharmacokinetic parameters parallel well with the short-lived psychoactive and physiological effects. No reports on toxicity or serious adverse outcomes were found. A variety of therapeutic applications have been proposed for *S. divinorum* which includes the treatment of chronic pain, gastrointestinal and mood disorders, neurological diseases, and treatment of drug dependence. Notwithstanding, there is still limited knowledge regarding the pharmacology and toxicology features of *S. divinorum* and salvinorin A, and this is needed due to its widespread use. Additionally, the clinical acceptance of salvinorin A has been hampered, especially due to the psychotropic side effects and misuse, turning the scientific community to the development of analogues with better pharmacological profiles.

## 1. Introduction

*Salvia divinorum* Epling and Játiva is a psychoactive Mexican mint used for centuries by Mazatec Indian shamans or *curanderos* of north-eastern Oaxaca, Mexico, for divinatory and religious purposes and physical healing [1,2]. Common vernacular names include *Sally D*, *Magic Mint*, *Diviner’s Sage*, *Mystic Sage*, *Purple Sticky*, *Lady Salvia*, or just *Salvia*. As Mazatecans believe that this herb is the reincarnation of the Virgin Mary, the term *Maria Pastora* is also used [3,4]. The first botanical description of the plant dates from 1963 [5]. However, it was only in the 1990s that *S. divinorum* was brought to the United States (US) and its psychoactive properties were recognised [2].

The main bioactive compound of this plant is salvinorin A, the first non-nitrogenous diterpenoid with psychoactive properties that was described, which was first isolated from the leaves of *S. divinorum* in 1982 [6]. It is one of the most potent natural hallucinogens known to date [7], with 200 µg being sufficient to trigger biological effects in humans after smoking [2]. From a pharmacological and chemical point of view, salvinorin A is quite unique due to two main features: (i) it is the only known non-alkaloidal hallucinogen, and (ii) it is the first naturally occurring non-nitrogenous compound that acts as an efficacious and potent κ-opioid receptor (KOP)-selective agonist [8,9,10,11]. Although not having affinity for the 5-HT_2A_ receptors known to mediate the hallucinogenic properties of the classic psychedelics, such as lysergic acid diethylamide (LSD), psilocybin [12], mescaline [13], and *N,N*-dimethyltryptamine (DMT) [14], salvinorin A induces similar psychoactive effects, including perceptual distortions, alterations in affect, behaviour, and cognition, and out-of-body experiences, with such effects being intense and short-lived [3,15]. The consumption of this substance has been associated with minimal or no health risks, thus being conceded as a safe hallucinogen [3].

The recreational use of this novel psychoactive drug has increased within the last 20 years, both in the US and Europe [16], mainly by adolescents and young adults [4]. In fact, it gained substantial popularity as a substitute for other common psychoactive substances, namely, cannabis derivatives and LSD [17], and through a search of YouTube videos it is possible to verify that *S. divinorum* and salvinorin A are becoming trendy topics [18,19]. Further fuelling this growing recreational use, leaf preparations of *S. divinorum* can be easily found and purchased over the Internet and in smartshops [3,20]. Seeds to be cultivated by the consumer; leaves that can be chewed, brewed, or dried for smoking; and liquid extracts used as a drink or smoked in water pipes are the forms under which the plant is normally sold [21]. Leaf materials can be sporadically found impregnated/fortified with salvinorin A extracts, enhancing the concentration of the substance and resulting in increased potency of the sold products (1–80x) [22]. Other factors contributing to its increased use are that *S. divinorum* enjoys legality in some countries, is apparently safe, and lacks detectability by commonly used drug screening tests [4]. The growing public representation of *S. divinorum* led to an increased mindfulness of its hallucinogenic properties, and apprehension about potential harmful outcomes of its use [23]. In fact, although *S. divinorum* is still an unregulated psychoactive plant in many countries, the United Nations Office on Drugs and Crime (UNODC) considered it a new psychoactive drug of concern [24].

Potential medicinal benefits of *S. divinorum* have long been recognised, mainly due to its use in folk medicine by the Mazatecans for the treatment of inflammatory disorders, rheumatism, headache, abdominal swelling, and diarrhoea [1,25]. Additional ethnomedical surveys also indicate its use for insect bites, eczema, candidiasis, and menstrual cramps [26]. An intricate and complex pharmacology and mechanism of action of this hallucinatory plant may be implicated in these several medicinal applications.

Herein, it is intended to comprehensively review the chemistry, pharmacokinetic and pharmacodynamic aspects of *S. divinorum* and its main psychoactive constituent, salvinorin A. With such a purpose, the psychological, physiological, and toxic effects induced by salvinorin A, as well as the available data on the pharmacology underlying possible therapeutic utility, will be covered. Information about its legal status, prevalence, patterns of use, and forensic relevance will be also included.

## 2. Methodology

An extensive English literature search was performed in PubMed (US National Library of Medicine) and Scopus, without a limited period of time, using the broad terms “salvinorin A”, “salvinorin”, and “*Salvia divinorum”*. Circa 321 articles were obtained. From these, approximately 64% dealing with the chemistry, pharmacokinetics, pharmacodynamics, toxicity, therapeutic potential, and forensic aspects of *S. divinorum* and salvinorin A or related compounds were identified and used for the purpose of this review.

## 3. Plant Botany and Chemical Characterisation of *S. divinorum*

**Figure 1:**
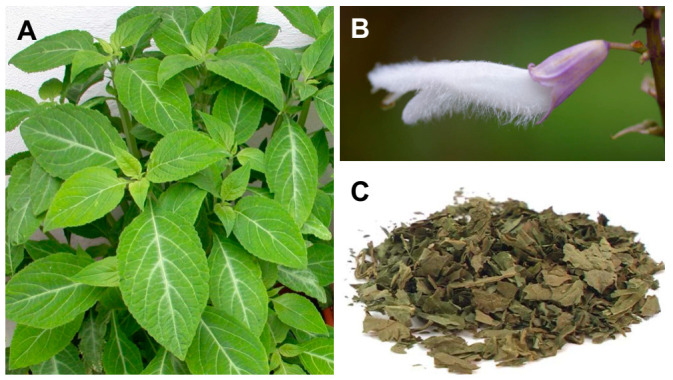
*Salvia divinorum* plant (**A**), the characteristic flower (**B**), and dry leaves (**C**).

*S. divinorum* is a perennial herb, endemic to the forest ravines of Sierra Madre Oriental of Oaxaca, Mexico [27]. Large green leaves and flowers with purple calyces and white corollas can be seen in *S. divinorum* plants (Figure 1) [3]. A maximum height of 1.5 m can be reached by these plants, and the leaves can be 10–25 cm long and 5–10 cm wide [5]. Cultivation of *S. divinorum* is usually accomplished at altitudes ranging between 750 and 1500 m, under hot, moist, humid subtropical environmental conditions [28]. *S*. *divinorum* is mainly widespread in Mexico and South American countries. Although the plant produces viable seeds, this is not frequent [5], with propagation being accomplished through vegetative reproduction.

*Salvia* spp. are known to be prolific producers of terpenes and terpenoids, particularly diterpenes, with the most common class of phytochemicals being reported in the genus [29]. Besides the main psychoactive constituent salvinorin A, other neoclerodane diterpenes have been isolated from the leaves of *S. divinorum*, which include salvinorins B–J, divinatorins A–F, salvidivins A–D, and salvinicins A and B [30,31,32,33,34,35]. Salvinorin A accumulates in large amounts on the glandular trichomes of the abaxial side of leaves [36]. Salvinorins were not observed in the roots, internal stem tissue, cotyledons, or corolla [36].

Concentrations of salvinorin A in the leaves of a single specimen can be extremely consistent over time [37]. However, different *S. divinorum* plants, even when genetically undistinguishable, can present considerable distinct concentrations in the leaves, varying from 0.89 to 7.8 mg/g of dried leaves. Table 1 displays salvinorin content in samples locally harvested or commercially obtained. The high variability in the amounts found might depend on the freshness of the leaves [38], geographic origin of the plant, cultivation technique [17,39], and adulteration of the traded products [40].

## 4. Biosynthesis, Structure, and Physicochemical Properties of Salvinorin A

The biosynthesis of salvinorin A occurs through the 2-C-methyl-erythritol 4-phosphate (MEP) pathway, involving class I and class II diterpene synthase (diTPS)-mediated reactions [42]. The precursor of this biosynthetic pathway was shown to be geranylgeranyl diphosphate (GGPP), which undergoes cycloisomerisation mediated by class II diTPS. The product of this enzymatic reaction is (–)-kolavenyl diphosphate, which is subsequently dephosphorylated by class I diTPS, rendering (–)-kolavenol. Regio- and stereoselective reactions further enable the production of a variety of intermediates and precursors of salvinorin A [42].

The total chemical synthesis of salvinorin A was first accomplished in 2007 through an asymmetric route [43] that featured a transannular Michael reaction cascade from a macrocyclic lactone, encompassing 29 reaction steps. The most recent advances in the topic were made by Wang and Metz [44], who developed a synthetic method involving only 18 reactions. Using two highly diastereoselective intramolecular Diels–Alder reactions as crucial transformations, salvinorin A was obtained from 3-furaldehyde. A comprehensive review on the total synthesis of salvinorin A was recently provided by Hill et al. [45].

**Figure 2:**
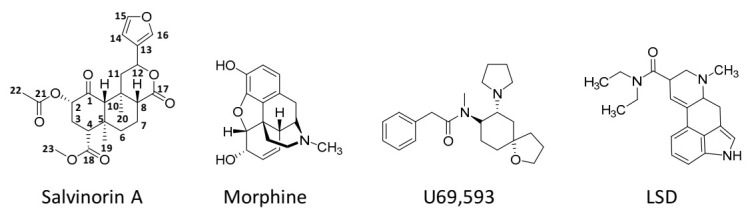
Chemical structures of salvinorin A, morphine (a well-known opioid), U69,593 (a potent and selective synthetic κ-opioid receptor agonist), and lysergic acid diethylamide (LSD, a recognised hallucinogen).

Unlike the other naturally occurring hallucinogens, salvinorin A (Figure 2) is a terpenoid that does not have nitrogen atoms in its molecular formulae (C_23_H_28_O_8_).

Pure salvinorin A is a colourless crystal with a high melting point, ranging from 238 to 240 °C. Salvinorin A is also distinguished from the majority of psychoactives as it is highly lipophilic [19], presenting limited solubility in water, with a logP (octanol/water) of 2.5 [46]. Soluble salts of salvinorin A cannot be formed due to the nonexistence of an ionisable functional group [47]. Salvinorin A has also low solubility in conventional safe vehicles applied in animal studies, with DMSO being commonly used instead [48]. It is worth noting that the high lipophilicity of salvinorin A enables passage through biological membranes, including the blood–brain barrier (BBB) [49].

## 5. Prevalence and Patterns of Use

*S. divinorum* is employed by natives of Sierra Mazateca in spiritual rituals and for therapeutic purposes [50]. Consumption ceremonies are performed during the night, in dark and remote locations to guarantee absolute silence and avoidance of distractions, lasting for 2–3 h [35]. Several restrictions are placed on participants, before and after the ceremony, so that they can reach a state of ritual purity. Amongst others, the most relevant limitations concern the prohibition of sexual activity, control over physical and verbal interactions, and the regulation of what attendants can eat and where they can go [50].

Results from a series of surveys indicate that teenagers, college students, and young adults are the main consumers of *S. divinorum*. Accordingly, based on the 2008 National Survey on Drug Use and Health (NSDUH), 1.8 million Americans aged 12 and older had used *S. divinorum* at least once in the past year [3]. According to the 2020 US annual Monitoring the Future (MTF) survey from the National Institute on Drug Abuse, conducted by mail and web on a representative sample of secondary students, college students, and adults, the overall prevalence of use has been decreasing since 2010 [51]. Between 2017 and 2019, consumption prevalence doubled to 0.8% in 8th graders (most students between 12 and 13 years old), with both college students and young adults (ages 19–28) seeing an increase in the prevalence of use from 2017 to 2018 (up to 0.9% in college students). The peak annual prevalence of *S. divinorum* use happened in 2009 for college students (5.8%), 2010 for the 8th graders (1.7%) and young adults (3.6%), and 2011 for 10th (3.9%) and 12th graders (5.9%) [51].

In a study performed during the fall of 2006 and spring of 2007 at a large public university in the southwestern US, 4.4% of the college population (predominantly Caucasian) had tried *S. divinorum* in the past year [52]. Participants also reported high co-consumption of marijuana (34.9%) and lower consumption of ecstasy (5.0%) and cocaine (7.1%) [52]. In a study by Khey et al. [53], who performed a questionnaire survey during the same period with undergraduates attending a public university in Florida, only 22.6% (190 students) had heard of *S. divinorum*, mainly from their friends (81.9%). Only 6.7% had lifetime use, 3.0% had used it in the last year, and 0.5% had used *Salvia* in the last month. Most users were male Caucasians, mainly from wealthy families. Most users (77%) reported buying *S. divinorum* from head shops, with half of them reporting intention to use the drug again [53].

Albertson and Grubbs [54] conducted a study during the 2007–2008 academic year, with 193 college-aged participants from a USA Midwestern university, collecting data on the demographic characteristics, history, and pattern of drug use. Users encompassed 34 individuals (57.6% male and 42.4% female), with an average age of 21 years. Eighteen volunteers reported consumption, which was every other month (83.3%), once a month (11.1%), or once a week (5.6%). *S. divinorum* was mainly obtained in head shops (58.8%), but also through a friend (35.3%) or was purchased from the Internet (5.9%). The reported consumption route was smoking the extract (60.6%), the leaves (21.2%), or both (18.2%), using a bong (55%) or a pipe (45%). None of the participants referred to religious or medicinal purposes as the motivation behind the consumption. Additionally, only 3% of consumers said they want to continue using *S. divinorum* on a regular basis [54]. In this study, it was further reported that users are more likely to consume more alcohol and cigarettes than non-users, and every user stated experience with marijuana (with nine individuals reporting mixing *S. divinorum* with marijuana).

In 2010, Baggott et al. [15] conducted an anonymous web-based survey of *S. divinorum* use, covering information on demographics, drug effects, and “when, how, and why” individuals had consumed this drug. A total of 500 participants were enrolled, primarily male (92.6%), with a mean age of 23 years (range 13–68), and most living in the US (77.4%). The mean lifetime use was 13.3 ± 22.9 days, use in the last year was 7.5 ± 11.6 days, and use in the last 30 days was 1.5 ± 2.6 days. The majority of consumers smoke or vaporise (92.6%) *S. divinorum*, either as a concentrated extract (61.4%) or dry leaves (37.3%). Only 4.2% used purified crystals of salvinorin A (by smoking or vaporisation [15]). Almost all participants (80.6%) reported they would probably/definitely use it again. The main motivations underlying the use were the desire to experience altered consciousness (86.2%), curiosity (76.6%), spiritual reasons (74.0%), personal growth or self-understanding (69.2%), contemplation or meditation (52.0%), relaxation or enjoyment (35.6%), or simply to get high (31.2%). Nyi et al. [55] also presented motivations for *S. divinorum* use by youth, stating that it was mainly for fun and to relieve tedium, while adults sought spiritual effects.

Cross-sectional data obtained from 42179 adolescents (12–17 years old) attending schools across Canada, who responded to the 2008–2009 Youth Smoking Survey, showed that 6.2% reported lifetime use of *S. divinorum* and 3.8% 12 month use [56]. First-time use occurred at a mean age of 14.6 years. Adolescents who had previously used cannabis or other hallucinogenic substances showed a higher probability of being lifetime users [56].

The consumption of *S. divinorum* has also increased in Europe since the late 1990s, mostly due to its Internet availability [57]. The 32 Spanish recreational *Salvia* users who participated in a study of González et al. [58] in 2010 reported, through self-assessment questionnaires, a preference for leaves and *S. divinorum* extract administration either through smoking (75%) or a combination of smoking and sublingual use (22%). The mean age of the respondents was 25 years, mostly male (56%). Regarding education, 72% of participants completed high school, 22% had a university degree, and 69% were attending university. Only 6% were unemployed. Participants obtained *S. divinorum* from “smartshops” (88%) or through a friend (12%). The history of other drug abuse substances was also assessed, with 93.7% reporting weekly alcohol consumption, 84.4% were smokers, and 96.9% were regular users of cannabis. They reported previous consumption of other drugs, including ecstasy (88%), cocaine (84%), amphetamines (69%), and opiates (56%). Psychedelic/hallucinogen consumption was also largely reported by 94% of the participants, of which psilocybin (78%), LSD (63%), ketamine (34%), and ayahuasca (28%) were the most frequent [58].

Few studies have evaluated the prevalence of *S. divinorum* consumption in Portugal. Those which have used small size samples or sampling was restricted to a specific group. During January and February 2013, an exploratory study with questionnaires was applied to 500 students from the University of Lisbon [59]. *S. divinorum* was amongst the new psychoactive substances most consumed, with 5.6% of students reporting its lifetime consumption, and 3.8% reporting consumption in the last 12 months. In 2014, questionnaires were applied to 30 students from the same university who were consumers of legal and/or illegal drugs [60]. A total of six individuals had already consumed *S. divinorum*.

In 2011, participants recruited by announcements posted on Facebook, online retailers, and Internet sites where *S. divinorum* or other drugs were mentioned, completed a questionnaire to collect their sociodemographic information and drug use history [23]. Of the 154 individuals who completed this survey, the majority were residents in the US (59.7%) or in the United Kingdom (18.8%), 83.1% were males, 16.9% were females, 87.7% were Caucasians, and the mean age was 25 years. Regarding professional occupation, 35.1% were students, 43.5% were employed, and a total of 38.3% had at least an undergraduate university degree. Considering *S. divinorum* use history, the first use was at 21 years old, and 73.4% said they consumed this drug in the previous year. These individuals most frequently obtained this drug from smartshops (85 users), online retailers (67 users), and friends or relatives (37 users). At least a third of the respondents (33.2%) used other drugs concomitantly with *S. divinorum* (1–2 h apart), with alcohol (13.7%) being the most reported, followed by cannabis (33.2%) and other hallucinogens (6.6.%) [23].

Overall, results from these studies support that consumption of *S. divinorum* is more prevalent amongst older adolescents/young adults and Caucasian males, with previous and concomitant use of marijuana, tobacco, alcohol, or other hallucinogens, and with higher total family income. While these younger individuals seek the drug for recreational purposes and use it predominantly in polydrug abuse settings, older users are mostly driven by spiritual and divination effects. Furthermore, prior involvement in risk-taking behaviours (e.g., selling drugs, stealing) and self-reported depression and anxiety were also related with increased probability of becoming a *Salvia* user [61].

## 6. Legal Status

*S. divinorum* has a somewhat indefinable legal status, contrary to most psychoactive substances. Indeed, the plant is not currently listed under the Controlled Substances Act [62]. However, it has been identified as a “drug or chemical of concern” by federal drug agents who have witnessed its growing use [63]. *S. divinorum* or salvinorin A have, in fact, been controlled in a number of individual US states, hampering research advances, namely, on the potential therapeutic benefits and biological effects [3]. Among these US states are, for example, Michigan [64], Missouri, Connecticut, Florida, Pennsylvania, Delaware, and Oklahoma [63,65,66]. In states like Georgia, Louisiana, Tennessee, and North Carolina, *S. divinorum* is legal but only when not intended for human consumption (e.g., as an ornamental plant) [66].

Australia was the first country that prohibited the possession, distribution, and selling of *S. divinorum* in 2002. In the following years, countries like Spain, Germany, Denmark, Belgium, Sweden, Poland, Italy, France, and Croatia placed *S. divinorum*, salvinorin A, or both on their lists of controlled substances, prohibiting possession and/or sale [66,67,68]. Notwithstanding, in Norway, Iceland, Finland, and Estonia, this plant can be legally used for medicinal purposes, including in the treatment of cocaine dependence [69,70]. In Portugal, Decree no 54/2013, 17 of April prohibited the production, distribution, sale, and possession of *S. divinorum*, salvinorin A and salvinorin B [71]. Following the implementation of the New Zealand 2014 Psychoactive Substances Amendment Act, all new psychoactive substances, which included *S. divinorum*, become legally controlled in this country [72].

Regarding Asian countries, Hong Kong, Japan, and South Korea have legislated *S. divinorum* and salvinorin A [70], but they are legal in India, Malaysia, Indonesia, and Thailand. In Mexico, there is no regulation on *S. divinorum* use or sale, although the Mexican government expressed the desire to include the plant and salvinorin A on the list of psychotropic substances of the Health and Safety Law [73]. *S. divinorum* and salvinorin A are also legal in some countries of South America (e.g., Colombia, Peru, Argentina), and in other European (e.g., the Netherlands, Austria) countries [74].

## 7. Pharmacokinetics

### 7.1. Routes of Administration and Absorption

Absorption of salvinorin A and other constituents of *S. divinorum* can occur through the oral mucosa and the pulmonary route, in the former to a lesser extent [67]. Some users also report insufflation of salvinorin A crystals [75,76], so absorption through the nasal mucosa should also be considered.

Consumption of *S. divinorum* by the Mazatecans is accomplished by chewing the fresh leaves or drinking the infusion or juice of an extract [3,25,58]. Thereafter, the onset of effects occurs at 10–15 min and they peak at 20–40 min, with the experience lasting for nearly 1 h [2,77]. When *S. divinorum* leaves are masticated, the juice must be retained in the mouth for approximately 10 min, before being spat out or swallowed, to enable the absorption through the oral mucosa of enough salvinorin A to produce psychoactive effects [2,15]. The percentage of salvinorin A being absorbed through the oral route in humans was reported to be around 85.8% [78]. Through sublingual administration of salvinorin A in a DMSO/PEG-400 vehicle, doses up to 4 mg exerted no psychoactive effects in eight volunteers [79], suggesting low bioavailability of salvinorin A through this absorption route. This may be due to the shorter period of time (5 min) that participants had to hold the liquid in the mouth. Immediately swallowing produces no psychoactive effects, even at doses as high as 10 mg, suggesting rapid and extensive enzymatic deactivation in the gastrointestinal tract or a significant first-pass metabolism [2].

On the other hand, recreational users consume *S. divinorum* sublingually or through inhalation following the smoking of dry leaves or volatilisation of the fortified extracts [3,25,58]. Vaporisation followed by inhalation of pure salvinorin A (>99%) seems to be an efficient route of administration, as indicated by the considerable levels of this compound in urine [80]. After inhaling the smoke of the dried leaves or the vaporised extract, this drug produces a rapid onset of effects and an intense high within 30 s, having a peak effect at 2–5 min after inhalation that lasts for 5–10 min and subsides over 20–30 min [2,21,58,68]. In humans, 250–500 μg of salvinorin A (equivalent to 3–7 μg/kg) is the minimum inhalation dose to enable hallucinogenic effects [8].

After inhalation of 1 mg of vaporised salvinorin A by volunteers, the drug reached a C_max_ of 31 ng/mL at 1–2 min, presenting rapid absorption that is coincident with reported times for the onset and peak of effects [80]. The area under the curve (AUC) varied from 370 to 487 ng/mL/min. Robust correspondence between plasma levels and subjective effects after volunteers’ inhalation of a single high dose of salvinorin A (18 or 21 μg/kg), was also previously observed [81]. The mean T_max_ occurred at 2 min post-inhalation, with plasma concentration of the drug rapidly declining to almost 0 at 90 min post-inhalation (final time point) [81]. In another study, plasma T_max_ was obtained approximately 15 min after inhalation of 8–12 mg of vaporised salvinorin A (approximately 114–171 μg/kg for an ordinary 70 kg human) by healthy volunteers [82].

The intravenous (i.v.) administration of salvinorin A has been described in animal models, including non-human primates [83]. Reports in humans have not yet been described. After i.v. administration of 32 μg/kg salvinorin A to rhesus monkeys, immediate sedation-like effects were observed, resolving in approximately 15 min. Toxicokinetic gender differences were observed, as in male subjects, the AUC was 572 ± 133 ng/mL/min, whereas in females it was 1087 ± 46 ng/mL/min [83].

Finally, in a study with Sprague Dawley rats intraperitoneally (i.p.) administered with a single high dose of 10 mg/kg salvinorin A, a rapid absorption was reported with a C_max_ of 345 ng/mL at 15 min [49].

### 7.2. Distribution

Salvinorin A is very lyophilic and therefore strongly binds to plasma proteins (binding in baboons was reported to be approximately 83%) [84]. A rapid biodistribution was observed in male rhesus monkeys (t_1/2_ value unknown) after i.v. administration of 32 μg/kg of salvinorin A, with this distribution being slower in females (t_1/2_ 0.95 ± 0.20 min), which suggests gender differences [83].

As expected, the drug easily and rapidly crosses the BBB [85]. Butelman et al. [86] documented a rapid entrance into the central nervous system (CNS) after i.v. administration of 32 μg/kg of salvinorin A to rhesus monkeys. Accordingly, the substance was detected in the cisternal cerebrospinal fluid (CSF) 1 min after injection, with maximum concentrations of approximately 1 ng/mL being recorded 2 min after administration [86]. Forty seconds after i.v. administration of [^11^C]-salvinorin A to six adult female baboons, the drug could be seen crossing the BBB and attaining maximum concentration in the brain, which corresponded to 3.3% of the administered dose [84]. Such results suggested that psychoactive effects in humans can be achieved with brain concentrations lower than 10 μg of salvinorin A (corresponding to a smoking dose of 200 μg of the drug) [84]. Accumulation in the cerebellum and visual cortex was reported, with both areas being intimately related with the hallucinogenic effects [84]. A similar distribution pattern was observed in the rat brain [87]. Such prompt entrance into the CNS occurs due to the high lipophilicity/permeability of salvinorin A, enabling it to passively diffuse through the BBB.

Hooker et al. [84] also evaluated the distribution pattern of [^11^C]-salvinorin A in peripheral organs of female baboons. Immediately after i.v. administration, the highest salvinorin A concentration was found in the kidneys (approximately 0.11% of injected dose per cubic centimetre). At 10 min after administration, salvinorin A began accumulating in the gallbladder, achieving its highest concentration of any organ at 60 min (approximately 0.12% of injected dose per cubic centimetre).

Clearance of salvinorin A from the baboon brain was also rapid, with elimination t_1/2_ determined to be 8 min [84], coherent with the short-term effects of the drug (less than 10 min). In less than 30 min after administration, concentration in the brain was down to 25% of the maximum determined. A gradual decrease in salvinorin A brain concentration was also observed in rhesus monkeys up to 30 min after administration. This is consistent with the duration of effects reported after consumption by humans, and with an active efflux mechanism from the brain. Along this line, studies with Madin–Darby canine kidney (MDCK) cells expressing MDR1 and with rhesus monkeys suggested that salvinorin A may be a substrate for P-glycoprotein (P-gp), a known key ATP-dependent efflux pump present in the BBB, kidney, and liver [49,88]. In a study with Sprague Dawley rats administered with a single i.p. dose of 10 mg/kg of salvinorin A, a maximum brain concentration of 23.9 ng/mL was obtained at 10 min, and the elimination t_1/2_ from this tissue was of 36.1 min [49]. Although a large volume of distribution was observed for salvinorin A (V_d_ = 47.1 L/kg), a low brain-to-plasma ratio was obtained (0.092–0.074 over 60 min), implying that a small proportion of the administered dose is effectively distributed throughout the organ [49].

### 7.3. Metabolism

**Figure 3:**
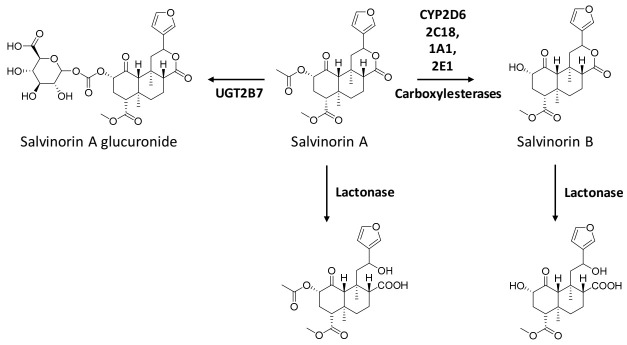
Metabolism of salvinorin A.

The metabolic pathways of salvinorin A are shown in Figure 3.

Salvinorin A is rapidly metabolised via ester hydrolysis at the 2-acetoxy moiety by blood esterases, mainly carboxylesterase, as demonstrated in in vitro and ex vivo non-human primate studies [89,90,91]. In vitro studies suggested that salvinorin A is also a substrate of a number of CYP450 isoforms, and it is worth emphasising the metabolism mediated by CYP2D6, CYP2C18, CYP1A1, and CYP2E1 [49]. Salvinorin B, the deacetylated form of salvinorin A, is the major representative metabolite. Since it is behaviourally inactive, this could explain the brief duration of action induced by salvinorin A [85]. Salvinorin B does not reach detectable levels in rhesus monkey plasma [83,89], raising speculations on its either rapid in vivo clearance or accumulation in the tissues.

Glucuronidation of the ester group at C2 by UGT2B7 was also observed, and was suggested to represent a major metabolic pathway of salvinorin A [49]. Both ester hydrolysis and the conjugation of salvinorin A were shown to display Michaelis–Menten kinetics, with it being saturable. Since these enzymes involved in the metabolism of salvinorin A are common to other drugs of abuse and pharmaceuticals, it is important to highlight the increased probability of pharmacokinetic drug–drug interactions.

Other metabolites of salvinorin A detected in vitro included the lactone ring open forms of salvinorin A and salvinorin B, which were suggested to be produced via the activity of calcium-dependent lactonase [90].

### 7.4. Excretion

The excretion of salvinorin A seems to occur through renal filtration for hydrophilic conjugated metabolites and biliary elimination for the remaining lipophilic metabolites [84].

Salvinorin A has a very short elimination half-life (t_1/2_) in vivo [92]. Following i.v. administration (32 μg/kg) to nonhuman primates, an overall elimination t_1/2_ was established at 56.6 ± 24.8 min [83]. Gender differences were also found in the elimination kinetics, with male monkeys having an elimination t_1/2_ of 37.9 ± 5.6 min, which was higher in females (80.0 ± 13.1 min) [83]. In another study, it was further determined that 5 min are sufficient to eliminate nearly 50% of a salvinorin A from baboon plasma, after i.v. administration [84]. The rapid elimination of salvinorin A is consistent with the short-term effects produced by *S. divinorum*. In Sprague Dawley rats administered with a single i.p. dose of 10 mg/kg salvinorin A, a rapid elimination from plasma was reported (t_1/2_ = 75.4 min) [49]. A plasma clearance of 26 L/h/kg was obtained.

After inhalation of 1 mg of vaporised salvinorin A by healthy volunteers, the drug was rapidly eliminated, with a t_1/2_ varying from 49–50 min [80]. In two human volunteers who smoked 75 mg of *S. divinorum* dry leaves (corresponding to 0.58 mg of salvinorin A), only approximately 0.8% of the dose was recovered from urine collected up to 1.5 h after consumption, with no salvinorin A being detected thereafter, which is consistent with a short half-life and extensive metabolism [68]. No salvinorin A was detected in sweat, which is coherent with the non-polar nature of salvinorin A [68]. Nevertheless, the low dose of salvinorin A being administered and the small volume of sweat sample collected might have also contributed to the observed results.

## 8. Drug Analysis and Forensic Relevance

The presumptive analysis of salvinorin A might be challenging as the compound and its metabolites are not detected in biological fluids by regular and broadly available, in situ drug screening tests [93], such as those used for police road inspections. Recently, a monoclonal antibody named 3D5 was developed to be used in indirect competitive enzyme-linked immunosorbent assays (icELISAs) for the detection of salvinorin A (Table 2) [94]. Several methanol extracts of *S. divinorum* and other species of the Lamiaceae family were analysed with the immunochemical method developed, with salvinorin A being only detected in *S. divinorum*, at concentrations varying from 9.72 to 16.7 μg/mg dry wt [94]. A few years later, the research group developed a new immunochromatographic assay (ICA) using the aforementioned monoclonal antibody combined with the icELISA, aiming at identifying salvinorins in *S. divinorum* [95]. The combined technique resulted in higher sensitivity, accuracy, and reliability of detection.

Conventional analytical methods for the quantitative analysis of salvinorins A and B include thin layer chromatography (TLC) and hyphenated chromatographic techniques, such as liquid chromatography–mass spectrometry (LC-MS) and gas chromatography–mass spectrometry (GC-MS).

A methodology based on GC-MS, following liquid–liquid extraction (LLE), was developed for the detection and quantification of salvinorin A in plasma, urine, saliva, and sweat [68] (Table 2). This methodology was applied to real samples, after two volunteers smoked 75 mg of *S. divinorum* dried leaves, containing a dose of 0.58 mg of salvinorin A. Concentrations of 2.4 and 10.9 ng/mL salvinorin A were found in 10 mL of urine collected 0–1.5 h after smoking, and 11.1 and 25.0 ng/mL in 2 mL of saliva collected 1 h after smoking [68]. Blood samples were not used since volunteers refused its collection, and salvinorin A could not be detected in sweat. Microextraction by packed sorbent (MEPS), coupled with GC-MS/MS analysis, allowed quantitative determination of salvinorin A in human urine [96]. A low sample volume (0.2 mL) was necessary to validate the method (Table 2). Salvinorin A was shown to be stable in the autosampler and in the samples, both at room temperature, for at least 24 h. Margalho et al. [97] developed and validated a gas chromatography–mass spectrometry with electrospray ionization (GC-MS/ESI) (Table 2) to quantify salvinorin A in the pericardial fluid (250 μL), vitreous humour (100 μL), whole blood (250 μL), and plasma (250 μL). At room temperature, salvinorin A remained stable in the processed samples for 3 h, and in the autosampler the stability was extended for 24 h [97].

An LC-MS/MS method in positive electrospray ionisation (ESI) mode, for the quantification of salvinorin A in non-human primate CSF and human plasma, was reported [98]. The parameters of method validation are compiled in Table 2. LC-MS-ESI, coupled to solid-phase extraction (SPE), was validated in urine samples for forensic analysis of salvinorin A [99].

It is worth referring to the development and validation of a method for the simultaneous detection of hallucinogens by ultra-high performance liquid chromatography—tandem mass spectrometry (UHPLC-MS/MS) in hair that detected 3.2 ng of salvinorin A per mg of hair in a 2 cm hair shaft from an *S. divinorum* consumer [100]. High-performance liquid chromatography–atmospheric pressure chemical ionisation mass spectrometry (HPLC-APCI-MS), coupled with SPE, was used by Schmidt et al. [89] to detect salvinorin A in a variety of biological specimens: human plasma and urine, and rhesus monkey plasma and CSF. The method was validated with human plasma (0.5 mL) spiked with salvinorin (8 and 40 ng/mL) (Table 2). The percentage of recovery from human urine and rhesus monkey CSF was higher than 102%, and that from rhesus monkey blank plasma was higher than 98%.

It was previously observed that the concentration of salvinorin A in incubated blood samples of rhesus monkeys rapidly decreased within 30 min, with an inverse increase in the amount of salvinorin B due to blood esterase-mediated metabolism [89]. It was thus suggested that salvinorin B, as the major metabolite in vivo, could be used as a concomitant target, along with salvinorin A, in newly developed and optimised analytical methods for the detection of human consumption of *S. divinorum* in different body fluids [38]. This could probably be applied successfully in clinical and forensic contexts.

Barnes and Snow [17] used LLE and solid-phase microextraction (SPME) to obtain salvinorin A from plants, aqueous solutions, and human urine. The SPME showed greater quantitative performance than LLE for the extraction of salvinorin A from water and urine samples [17]. Detection of salvinorin A was then performed with comprehensive two-dimensional gas chromatography–time of flight mass spectrometry (GC × GC-ToFMS), revealing the presence of salvinorin A in the leaves and stems, but not in the roots. GC × GC-ToFMS also enabled the separation of the related analogues of salvinorin A, with salvinorin B and C being detected in the leaves. Although the required volume of urine was high (20 mL), adequate limit of detection (LOD) (5 ng/mL) and limit of quantification (LOQ) (8 ng/mL) were obtained.

The identification of *S. divinorum* and its distinction from other plant materials, such as foods and medicinal herbs, is oftentimes difficult, contributing, to some extent, to the unregulated state of the drug [101]. Direct analysis with real-time high-resolution mass spectrometry on headspace volatiles sampled using solid-phase microextraction (SPME) fibres enabled the rapid detection and identification of plant-based legal highs, including *S. divinorum*, allowing the creation of a robust database [101]. Nevertheless, the identification of *S. divinorum* in forensic laboratories is often accomplished through the disclosure of the presence of salvinorin A in the plant materials.

TLC-GC/MS was used to analyse five commercially available leaves and extract products of *S. divinorum* [40]. Concentrations determined in all samples were from 1 to 16% of those stated on the labels, and not coincident with the potency reported (e.g., a sample labelled 1× had higher levels of salvinorin A than a sample labelled 5×). As such, users are exposed to increased risk of overdose as a result of the incongruities between the salvinorin A concentration advertised on the product’s label and that actually observed. Moreover, caffeine and vitamin E were found as adulterants in three analysed samples. Combinations of TLC with desorption electrospray ionisation (DESI)-MS [102] and HPLC-UV-VIS [103] were shown to be feasible for the detection and quantification of salvinorin A through the analysis of untreated, dry *S. divinorum* leaves of commercially sold plant samples.

A HPLC method to quantify salvinorin A in *S. divinorum* products sold throughout Mexico was established and validated [73], ascertaining that salvinorin A concentration in the products (ranging from 8.32 to 56.52 mg/g of dry leaf) did not correspond to the amounts expected for each strength, nor to the amounts claimed on the packaging tags. Moreover, there was no consistency in salvinorin A content between different brands [73]. The same inconsistencies and mismatching with labelled concentrations were reported in previous studies that used HPLC-UV, LC-MS, and GC-MS to analyse *S. divinorum* extracts [22,104].

Xavier Moreira et al. [104] validated a GC-MS protocol (Table 2) for the quantification of salvinorins A–D in concentrated extracts of *S. divinorum*. Ten different products purchased from eight Portuguese smartshops and two Internet websites were tested, with potencies varying from 5x to 60x. Salvinorin D was present in residual amounts in the samples, all below the LOQ of the method, preventing quantification. The concentrations of salvinorin A in the products ranged from 2.6–521.2 mg/g, and it was possible to verify an increase in the drug content with the increase in the labelled potency [104]. Most of the products did not provide information on the label regarding the content of salvinorin A, but for those which did (five products), salvinorin A concentrations were lower than what was labelled for three products, and concentrations were slightly higher than expected for the other two products. Salvinorins B and C were only detected in some of the samples analysed, and their concentrations ranged from 3.08–117.86 mg/g and 2.91–11.87 mg/g, respectively.

Similar to other so-called herbal highs, commercialised powder of *S. divinorum* can be simply adulterated by the addition of other plant species in the form of dried leaves. Therefore, it is imperative to develop analytical tools to correctly identify the constituents of these plant matters. Morphological, anatomical, and chemical characteristics of plants are general approaches for this identification, however, a variety of limitations to their application can be pointed out, like the samples’ physical status and the environmental factors during plant development [37]. A new approach, using molecular genetic methods, was presented by Bertea et al. [37]. Rapid and accurate identification of *S. divinorum*, in the form of fresh or dried leaves and powdered materials, was accomplished using a combination of chemical (HPLC-MS) and molecular (DNA fingerprinting based on the 5S rRNA spacer region sequence) analytical techniques, demonstrating its feasibility for application in forensic and toxicological investigations [37]. Murphy and Bola [105] also used DNA approaches, specifically the sequencing of the *rbcL* gene amplified with PCR, to distinguish between *S. divinorum*, *Cannabis sativa*, and *Nicotiana tabacum*. Additionally, distinction from other *Salvia* species (e.g., *S. chionophylla*, *S. microphylla*, *S. clevelandii*, etc.) was also accomplished [105].

## 9. Pharmacodynamics

Salvinorin A lacks interaction with the 5-HT_2A_ receptor, the characteristic target of classical hallucinogens (e.g., LSD, psilocybin, mescaline, DMT) [2]. Instead, the drug acts as a selective, highly efficient KOP total agonist, binding with an affinity of K_i_ = 16 nM and a potency of EC_50_ = 1.2 nM [10,11], through hydrophobic interactions with both conserved and non-conserved amino acid residues of the receptor [38,106,107].

Based on structure–activity relationship studies, it was verified that the size and nature of the C2-substituent moiety of salvinorin A are critical for the efficacy and potency of KOP agonistic activity [11], with the C2-acetyl group being crucial for its high affinity [108]. The β-hydrogen at the C8 position is also of structural importance for the drug’s psychoactivity [109].

**Figure 4:**
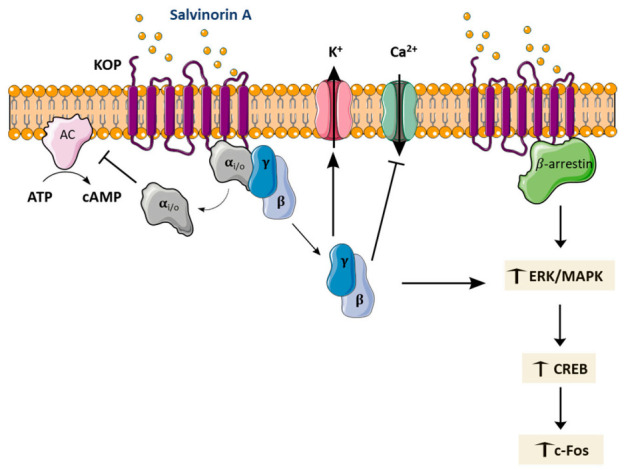
Schematic representation of the downstream pathways triggered by κ-opioid receptor (KOP) activation by salvinorin A. Following KOP activation, the Gα subunit of Gi/o proteins dissociates from the Gβγ subunit, with these consequently interacting with intracellular effectors [114]. The production of cyclic adenosine monophosphate (cAMP) by adenylyl cyclase (AC) is inhibited and causes the activation of inward-rectifying K^+^ channels, and inhibition of N-type, P-type, Q-type, and R-type voltage-activated Ca^2+^ channels, leading to an inhibitory effect on neurotransmitter release [115,116]. Additional downstream signalling pathways involve the activation of β-arrestin and phosphorylation of extracellular signal-regulated kinases (ERKs)/mitogen-activated protein kinases (MAPKs) by Gβγ subunits. This leads to activation of cAMP response element-binding protein (CREB), and a consequent increase in c-Fos expression. ATP: Adenosine triphosphate.

Opioid receptors can be mainly subdivided into μ-opioid (MOP), δ-opioid (DOP), KOP, and nociceptin-opioid (NOP) receptors, which are hepta-helical receptor members of the G-protein coupled receptor superfamily. KOP receptors are encoded by the *OPRK1* gene [110], and its activation triggers a signalling pathway involving the G-protein subfamily Gi/o (Figure 4), whose stimulation produce analgesic and/or psychotomimetic effects [27]. KOP receptors are found in the hippocampus, hypothalamus, striatum, amygdala, and spinal cord [28], areas associated with learning and memory, emotional/stress control, and pain. Many physiological functions are believed to be affected by the modulation of this receptor, including pain relief, depression, anxiety, stress, and psychotic symptoms [111]. Salvinorin A appears to be more effective than common KOP agonists [112], which might be due to the fact that interaction with the receptor occurs through a different binding configuration, as salvinorin A does not have an ionisable amine moiety [112,113].

The affinity of salvinorin A to KOP has been demonstrated in drug discrimination studies with rhesus monkeys [117] and rats [19,118] trained to recognise the stimulus cue induced by known synthetic κ-agonists, i.e., U69,593 and U50,488. A full substitution with salvinorin A was seen, with these results being blocked by KOP antagonists administrated prior to salvinorin A. In rhesus monkeys [117], similar dose- and time-dependent effects of salvinorin A and U69,593 were produced, suggesting analogous stimulant properties. Ansonoff et al. [48] delivered evidence supporting that the antinociceptive and hypothermic effects of salvinorin A in mice are KOP-1 dependent, and not related to the KOP-2 subtype whose mechanism was suggested to involve DOP interaction [119]. Opioid receptor subtypes such as KOR1 and KOR2, as well as their equivalence to homo- or heteromers, remain, however, largely debated.

Additional downstream pathways can take place after KOP activation by salvinorin A, including β-arrestin 2-mediated signalling and extracellular signal-regulated kinase (ERK)/mitogen-activated protein kinase (MAPK) pathways (Figure 4), which can be responsible for the side effects arising from KOP activation (e.g., dysphoria, sedation, aversion) [114,115,120].

Compared to other known potent synthetic KOP agonists (e.g., U50,488, U69,593, TRK-820), salvinorin A is not an ordinary ligand, as it promotes less internalisation of the human KOP (40-fold less potent) and less downregulation of the membrane receptors [121]. Recently, KOP activation and internalisation through the β-arrestin signalling pathway have been shown to mediate the salvinorin A protective action towards oxygen–glucose deprivation-induced cell injury, through inhibition of autophagy [122].

After acute and repeated (at days 1–5, 8, and 9) i.p. administration of 2 mg/kg of salvinorin A to Sprague Dawley rats, ERK1/2 phosphorylation increased in the nucleus accumbens, accompanied by the activation of cyclic adenosine monophosphate (cAMP) response element-binding protein (CREB), which is an ERK substrate [123]. An induction of c-Fos in the nucleus accumbens was also observed in this study, but only after acute administration of salvinorin A (Figure 4).

A few studies also evaluated the capacity of salvinorin A for substitution in animals trained to discriminate other hallucinogens. In rhesus monkeys, salvinorin A was not able to substitute for the serotonergic hallucinogen 2,5-dimethoxy-4-methylamphetamine (DOM) [124], suggesting different pharmacodynamic interactions. In Sprague Dawley rats trained to discriminate LSD and ketamine, a non-generalisation to salvinorin A was verified [125], also demonstrating that the psychoactive effects produced by the drug are different than those produced by the classic hallucinogens and, therefore, suggesting different pharmacodynamic mechanisms.

**Figure 5:**
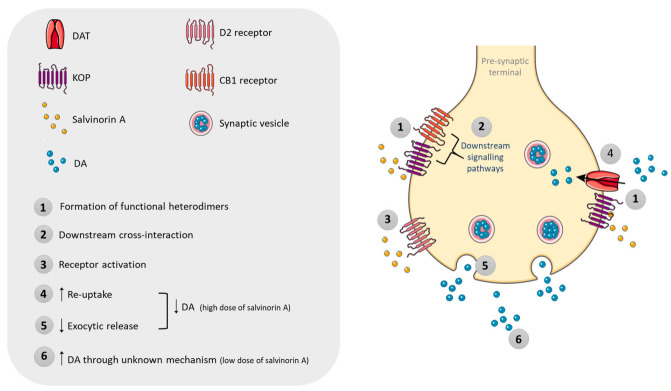
Proposed salvinorin A interaction with the endocannabinoid and dopaminergic systems. Of note, the represented mechanisms are entirely speculative [86,108,127,135], alternative explanations for the impact of salvinorin A on dopamine (DA) levels, including the binding of the drug to the κ-opioid receptor (KOP) in the ventral tegmental area, reducing DA release in the striatum and the nucleus accumbens. CB1 receptor: Cannabinoid receptor type-1; D2 receptor: Dopamine receptor type-2; DAT: Dopamine re-uptake transporter.

Besides KOP, several studies suggest that effects of salvinorin A in the CNS and at the periphery involve cannabinoid receptors [108,126,127,128,129], since some of these effects were significantly blocked by pre-treatment with either a KOP or a CB1 receptor antagonist. Despite that, salvinorin A does not display strong affinity towards CB1 nor CB2 receptors, not binding nor activating them [10,11,130]. As such, it was hypothesised that a downstream cross-interaction between KOP and CB1-mediated pathways [86,127] or the formation of functional heterodimers could underlie these effects [108] (Figure 5). These assumptions await experimental confirmation.

There is low consensus about the role of salvinorin A on the dopaminergic system (Figure 5). Nevertheless, salvinorin A was reported to interact and activate type-2 dopaminergic (D2) receptors in Sprague Dawley rat striatal tissue [131]. Activation of KOP has been associated with dysphoric effects in humans. Consistent with these effects, administration of salvinorin A (1.0 and 3.2 mg/kg, i.p.) to C57BL/6J mice resulted in a long-lasting, dose-dependent decrease in extracellular dopamine (DA) levels, for the low (30% decrease) and high (70% decrease) doses, in the caudate putamen [132]. Carlezon et al. [133] showed, in Sprague Dawley rats administered with salvinorin A (0.125–2.0 mg/kg, i.p.), a dose-dependent decrease in dopaminergic activation in the nucleus accumbens, the major brain structure involved in euphoria. Gehrke et al. [134] showed that acute systemic administration of salvinorin A (1.0 or 3.2 mg/kg, i.p.) to Sprague Dawley rats resulted in decreased dialysate extracellular DA levels in the dorsal striatum. Based on neurobiological studies, the decrease in extracellular DA may result from potentiation of DA re-uptake transporter (DAT) function by salvinorin A, which may be secondary to the formation of KOP/DAT heterodimers [135]. Other authors suggest that the pharmacological action is based on decreased DA release, without affecting the neurotransmitter uptake [134]. Conversely, small doses of salvinorin A (5–40 μg/kg, subcutaneous (s.c.)) led to the enhancement of DA dialysate levels, through a mechanism that is still not completely understood [129,136].

Experiments conducted with HEK 293 and EM4 cells exposed to salvinorin A (0.1–10 μm), showed a rapid upregulation of DAT function, an effect induced by KOP activation through an ERK1/2-dependent mechanism [135]. Immediate increased surface expression of DAT suggested enhanced trafficking of this receptor from intracellular compartments to the cell membrane. Furthermore, it was demonstrated that KOP and DAT are in close proximity, enabling them to interact. Accordingly, levels of DAT–KOP heterodimers increased following salvinorin A exposure [135]. Of note, heteromerisation between KOP receptors and DAT has not been established in vivo and therefore remains entirely speculative.

Salvinorin A (0.1–1000 nM) augmented the release of noradrenaline (NA) and inhibited the release of serotonin (5-HT) and DA from synaptosomes isolated from mouse hippocampus, striatum, and prefrontal cortex [137], with these effects on neurotransmitters release being mediated by presynaptic KOP activation.

Salvinorin A has an apparent negligible affinity for a variety of other receptors, transporters, and ion channels, namely, the MOP and DOP receptors, muscarinic and nicotinic cholinergic receptors, and several ionotropic and metabotropic glutamate receptors [10,138]. However, as evaluated in Chinese hamster ovary cells, salvinorin A was shown to allosterically modulate MOP receptor binding [139].

## 10. Psychological and Physiological Effects

### 10.1. Effects in Humans

The short-term effects of *S. divinorum* vary widely from person to person, and include modification of visual perception, hallucinations, out of body experiences, altered states of self and reality, dizziness, light headedness, disorientation, mood and somatic sensations, confusion of senses (e.g., hearing colours or smelling sounds), dysphoria, and increased vigilance [2,58,140]. Through observation of YouTube videos of “tripping” events, Lange et al. [141] stated that the behaviours appeared to be dose dependent and individualised. For new users expecting effects resembling those of marijuana, the rapid onset and intensity of *S. divinorum* can be disorienting [3]. An “acute positive experience” was the most common experience reported by youth, namely, improved mood (44.8%) and a feeling of calmness (42.6%). Positive effects lasting more than 24 h after use were also described by 25.8% of individuals, with improved mood being described as the main effect (46.5%) [55].

Behavioural, subjective, cognitive, and endocrine effects resulting from the inhalation of low (8 mg) and high (12 mg) doses of vaporised salvinorin A (administered daily for three days) were investigated in a double-blind, randomised, placebo-controlled, crossover, counterbalanced study involving ten healthy volunteers, with previous experience with *S. divinorum* consumption [82]. Transient psychotomimetic effects, somaesthetic alterations, dissociative effects, and perceptual alterations were induced by the drug, in a concentration-independent manner. No euphoria, cognitive deficits, changes in vital signs (heart rate and systolic/diastolic blood pressure), or adverse events were reported. Regarding the endocrine effects, salvinorin A significantly elevated cortisol and prolactin plasma levels, which returned to control levels 60 min after inhalation [82].

Maqueda et al. [142] investigated the subjective effects of salvinorin A in a double-blind study, involving eight healthy volunteers with previous psychedelic experience. During a 4-day study, participants daily self-administered one of three doses of salvinorin A (0.25, 0.50, and 1 mg) or placebo through inhalation after vaporisation, in a randomised sequence. Induced effects were dose dependent, with a peak at 2 min and lasting only 20 min. Volunteers experienced a sense of complete loss of contact with the body/environment and decreased body awareness following the high dose, with increased somatic bodily sensations at lower doses. Auditory sensations were common at all doses, with visions occurring in the medium and high doses. Feelings of calm and relaxation, leading to mood improvement, and a lack of negative aftermaths were also reported [142].

Disruption in vestibular and interceptive signals, such as pressure on the body and change in spatial orientation, as well as repeating childhood memories, contact with entities, and seeing cartoon-like imagery, were reported by four healthy hallucinogen-experienced adults after salvinorin A consumption through vaporisation (doses increasing from 0.375 to 21 μg/kg), in a double-blind, placebo-controlled study [21]. Additionally, a safe physiological profile under the study’s controlled conditions was reported, since no significant effects on blood pressure or heart rate, and no resting or kinetic tremors, were perceived [21]. A lack of dysphoric effects and dose-dependent positive effects was additionally reported.

A qualitative study based on email interviews showed that the subjective effects of *S. divinorum* were comparable with those produced by ketamine [77]. However, González et al. [58], through a quantitative study of 32 Spanish *Salvia* consumers, had 63% of participants reporting that the effects were analogous to other drugs of abuse, including psilocybin (55%), ayahuasca, ketamine, LSD, and marijuana (20% each). In another study with 39 *S. divinorum* users, the psychoactive experience was rated as “intense” to “extremely intense”, with the majority stating that it was more similar to a marijuana experience, with fewer reporting similarities with psychedelic mushrooms and LSD [54]. Some even reported that the *S. divinorum* experience was “too unique to be comparable” [54].

Addy [143] performed a double-blind, placebo-controlled, randomised study with 30 volunteers with a history of hallucinogen consumption, aiming to assess the acute and post-acute (at day 56) behavioural and psychological effects provoked by the smoking of salvinorin A. This study involved two sessions conducted at a 2-week interval, followed by a semi-structured open-ended interview, 8 weeks after the second session. Participants received both the active dose (25 mg dried *S. divinorum* leaves spiked with 1017 μg salvinorin A) and the placebo (presumed non-psychoactive dose of 25 mg unenhanced dried *S. divinorum* leaf, containing approximately 100 μg salvinorin A), in an aleatory way (one in each session). Blood pressure, temperature, pulse, and respiration rates were measured at baseline (10 min before) and at the end of the experience (mean 70 min after), to assess volunteers’ vital signs. The participants’ observable behaviour during the salvinorin A inebriated state was recorded through the Monitor Rating Questionnaire, beginning 30 min post-smoking. To assess the subjective experience, participants also completed the Hallucinogen Rating Scale (HRS), which comprises somaesthesia, affect, perception, cognition, volition, and intensity clusters. No significant alterations were observed in vital signs or perceived behavioural effects, including increased talking, laughing, movement, and fear, with no reports on sneezing, vomiting, goosebumps, or unresponsiveness. The ratings of all six clusters of subjective effects, as evaluated by the HRS, were increased after self-administration of the salvinorin A active dose. At the 8-week follow-up interview, volunteers (66.7%) reported aftereffects lasting less than 24 h after the session, with the positive effects (e.g., reflection, empathy, awareness of beauty) being more commonly reported than the negative ones (e.g., headache, fatigue, difficulty concentrating) [143]. In a follow-up article of this study, it was additionally stated that the experience of smoking salvinorin A was intense, unique, and had a rapid onset [144]. Affect, cognition, interception, and sense of reality, with participants losing normal awareness of themselves and their environment, are markedly changed after inhalation.

Karam et al. [145] performed a cross-sectional study in patients attending the Psychiatric Hospital of the Cross, Lebanon, between January and May 2016, aiming to assess various aspects of *Salvia* consumption through questionnaires. The mean duration of subjective effects reported by users was 15.52 min, with a rapid onset. By applying the HRS questionnaire, the only subscales showing a significant difference between *S. divinorum* users and non-users were intensity and volition (higher scores for non-user controls) and perception (higher scores for users) [145].

Studies in experienced hallucinogen users showed that although robust hallucinations, depersonalisation, and derealisation effects were felt, neither robust dysphoric nor aversive subjective effects were perceived after salvinorin A self-consumption [21,82,143,144,146].

### 10.2. Effects in Animal Models

In animals, KOP-mediated effects of salvinorin A at the central level include alterations at the cognitive [147] and behavioural [148] levels. Accordingly, behavioural conditional place aversion, sedation/motor incoordination, anxiety, nociception or antinociceptive effects, and depressive-like symptoms were amongst the behavioural effects detected in studies with rodents [126,132,133,149]. Neurobehavioral effects of salvinorin A have been studied in a variety of animal models, such as zebrafish, rodents, and rhesus monkeys. In mice and rats, salvinorin A induced sedation and decreased motor coordination (2 mg/kg, i.p.) [149], increased immobility, and decreased swimming, when used for assessing antidepressant-like behaviour (0.125–2.0 mg/kg, i.p.) [133]. After intramuscular (i.m.) injection of salvinorin A into the caudal musculature of zebrafish, at doses ranging from 0.1 and 80 μg/kg, a stimulant swimming behaviour was witnessed at low doses, while depressant effects occurred at higher doses. These effects were greatly blocked by pre-treatment with both KOP (nor-binaltorphimine) and CB1 (rimonabant) receptor antagonists [128].

There is no consensus on the central actions of salvinorin A. While pro-depressant-like effects were observed in Sprague Dawley rats (0.125–2.0 mg/kg, i.p.) [133], in a study with adult Sprague Dawley rats and Swiss mice given s.c. salvinorin A (0.001–1000 μg/kg), evidence of anxiolytic and antidepressant properties was obtained [150]. Sedative-like effects, reinforcing a depressant activity on the CNS, were observed after i.p. administration of non-polar, medium polar, and polar crude extracts of *S. divinorum* (10, 30, and 100 mg/kg) to Swiss albino mice [151]. After administration of the medium polar extract (10 mg/kg and 100 mg/kg, i.p.) to Wistar rats, sleep disruptions were induced, supporting a possible role of salvinorin A in the stimulus of sleep behaviour disorders. Harden et al. [152] observed that salvinorin A administration (1 mg/kg) reversed chronic mild stress-induced anhedonia in Long–Evans rats, agreeing with the alleged antidepressant properties of the drug. No depressant or antidepressant effects were observed when no stress-induced anhedonia was settled [152].

The antinociceptive effects of salvinorin A have been widely evidenced through a plethora of experiments (e.g., hot plate test, tail flick test, acetic acid abdominal constriction) [92,153], but contradicting results on the analgesic effects of the drug have also been attained [121], rendering antinociceptive properties of salvinorin A inconclusive [38]. After i.p. (0.5–4.0 mg/kg) and intrathecal (11.6–23.1 nmol) administration to mice, salvinorin A presented dose-dependent and short-lived analgesic effects [92,153]. Sedation and antinociceptive effects in mice were transient (maximum at 15 min and no effect at 45 min) following an intracerebroventricular injection of 7.5 μg of salvinorin A, while hypothermia was longer lasting (maximum at 75 min and no effects at 120 min), but only at 50 μg [48]. In mice injected with salvinorin A (0.56 and 1.8 mg/kg i.p.), a dose-dependent decrease in self-grooming was reported [154]. At the peripheral level, gastrointestinal function seems altered [108,127,155,156,157,158] by KOP-mediated inhibition of contractility due to a decrease in enteric cholinergic transmission [157]. Inhibitory activity on hypermotility induced by intestinal inflammation was also observed [127,158]. Aviello et al. [159] investigated the effect of pre-treatment with salvinorin A (0.01–10 pM, 30 min) on lipopolysaccharide (LPS) insult in murine macrophages, observing a reduction of LPS-stimulated nitrite (synthesised in response to inflammatory stimuli), tumour necrosis factor-α (TNF-α), and interleukin-10 (IL-10) levels, in a concentration-dependent manner [159].

A dose-dependent increase in prolactin release mediated by KOP was reported after i.v. administration of salvinorin A (3.2 μg/kg) to rhesus monkeys [110], with this effect being more evident in female than in male rhesus monkeys. Dose-dependent sedative and postural effects were also observed in the same animal model exposed to salvinorin A (32–100 μg/kg, i.v.), with a rapid onset (5 min) and short duration (15 min) [86]. These effects were prevented with the use of a KOP antagonist but not with a CB1 receptor antagonist. Additionally, facial relaxation and ptosis were observed (10–32 μg/kg of salvinorin A), which were rapid (onset 1–2 min after i.v. administration) and dose dependent [86].

Morani et al. [160] evaluated the aversive effect, the spontaneous locomotor activity, and the depression-like effects of salvinorin A, using behavioural conditional taste aversion and forced swim tests, after acute exposure to the drug (0.3 mg/kg, i.p.) in Sprague Dawley rats. At this dose, the authors observed an attenuation of cocaine behavioural sensitisation, modulation of cocaine-induced locomotor activity, with increase hyperactivity, and pro-depressive effects. However, decreased hyperactivity was reported in another study with Sprague Dawley rats, after acute administration of higher salvinorin A doses (2 mg/kg, i.p.) [148].

Many of the controversial properties of salvinorin A regarding its aversive/rewarding effects, and the pro-depressant/antidepressant effects, can possibly be explained by the different settings of the studies (e.g., dose and time of exposure, route of administration, animal model) [161]. Nevertheless, in general, there is a lack of understanding of these effects and associated mechanisms.

## 11. Toxicity and Adverse Effects

### 11.1. Effects in Humans

There is a lack of scientific evidence on the toxicity of *S. divinorum* [53]. There are no reports on acute or chronic toxicity induced by *S. divinorum* consumption, the occurrence of symptoms requiring treatment in an emergency department, or deaths from overdose [4,70,162]. On one hand, this can be due to its relative safety, but, on the other hand, clinicians may have been failing to recognise *S. divinorum* consumption by patients arriving at medical care units [4].

Tiredness, confusion, drowsiness/lethargy, heaviness of head, tachycardia, and dizziness are common adverse symptoms reported by *S. divinorum* consumers [15,58,163]. Other particularly negative outcomes can be strictly associated with salvinorin A, including fear, panic, paranoia, agitated delirium, sadness, irritability, augmented perspiration, and chills [55,67,128]. Consumers can feel a lack of insight, leading them to be susceptible to harm [162], which can occur during the performance of complex tasks (e.g., driving). Gastrointestinal malaises (e.g., nausea and vomiting) have also been reported [164]. The increased occurrence of side effects can be associated with either the high drug doses administered or with the concomitant consumption of large amounts of alcohol [54] and other drugs, such as hallucinogens, leading to an increased risk of developing neurologic, cardiovascular, and gastrointestinal complications [165]. Unfortunately, there is scarce information on the interactions between these (il)licit drugs and *S. divinorum* [73].

Following salvinorin A consumption, no evidence of continuing psychotic-like episodes or other negative effects (e.g., depression, anxiety) were found in laboratory research [82,143,146]. Contradictory studies and case reports state that salvinorin A users present higher rates of depression [16], and there is a possible association with the use of the drug and the precipitation of anxiety symptoms and acute clinical manifestations characteristic of psychotic disorders [93]. Currently, there is little evidence that suggests long-term consequences by *S. divinorum* [23,58], although impairment of memory and cognitive performance was related with chronic consumption [166].

Bad trips were also reported for *S. divinorum*. In a study by Baggott et al. [15], a participant reported the need to seek medical treatment due to a stimulant psychosis, after using *S. divinorum* along with DMT, dextromethorphan, methamphetamine, and diphenhydramine. A terrifying acute episode experienced by a man in his thirties after smoking *S. divinorum* dried leaves was also described [167]. The experience encompassed short-lasting (12–14 min) vivid visual and auditory hallucinations, including a female presence in the room, inanimate objects coming alive and talking, and ghosts talking to him, leading to feelings of extreme fear. However, no long-term perceptual disturbances were described by the individual [167]. No long-term cognition damage, significant tremor, blood pressure or heart rate changes, or continuing adverse effects were induced by salvinorin A consumption in humans at doses varying from 0.375–21 μg/kg [21,146], 114–171 μg/kg [82], and 15 μg/kg [143], suggesting that it can be safely consumed, especially when used in a supportive and supervised medicinal context. Nevertheless, increased blood pressure was observed in 24 volunteers following vaporisation of 1 mg (corresponding to 14 μg/kg for a 70 kg human) of salvinorin A [80]. Compared to other studies in which no cardiovascular effects were seen, this difference may result from the distinct time points at which measurements were taken, and the distinct efficiency of the vaporisation methods used, leading to different salvinorin A bioavailability.

A total of 37 cases related with recreational use of *S. divinorum*, 18 of which involved the sole consumption of this substance, were reported to the California Poison Control System from January 1998 to May 2008 [165]. From these cases, disorientation, agitation, hallucinations, dizziness, and flushed sensation were the most prevalent signs and symptoms. In two cases, abnormalities in vital signs were recorded and included tachycardia and a combination of tachycardia and hypertension. No deaths were attributed to *S. divinorum*. However, it is worth noting a single case of suicide after *S. divinorum* consumption [40].

A case report of a young woman who had a psychotic episode after smoking *S. divinorum*, which was added to a marijuana cigarette without her knowledge, was described by Paulzen and Grunder [168]. Several of her following medical complications appeared, however, to be more correlated with the medical treatment than the drug itself. Two other studies reported the onset of acute psychotic-like reactions (e.g., paranoia, *déjà vu*, slow speech) or persistent psychosis after *S. divinorum* consumption by two individuals aged 15 and 21 [169,170]. There were confounding factors for the correct interpretation of these cases, namely, co-consumption with other drugs [169] and an alleged genetical predisposition for schizophrenia [170]. A psychotic episode suffered by a 23-year-old male was reported, after consumption of an unknown dose of *S. divinorum* [171]. Previous personal or family history of mental/personality disorders was not documented, and there was no reported co-consumption with other substances. This psychotic episode was nevertheless rapid and easily manageable in an impatient psychiatry service, and no long-term mental effects were found in the 9 months of follow-up [171]. Based on the results of all studies analysed, *S. divinorum* and salvinorin A are not expected to induce flashbacks.

### 11.2. Effects in Animal and In Vitro Models

At doses greater than those used by consumers, salvinorin A (0.4–6.4 mg/kg, i.p.) had relatively low toxicity after acute administration to rats and chronic, 2-week administration to mice [172]. Interestingly, there was a slight, insignificant increase in pulse pressure after salvinorin A exposure, but no effects on respiratory rate, cardiac conduction, temperature, or galvanic skin response were observed. Histological analysis revealed no negative pathological changes in the spleen, blood, brain, liver, kidney, or bone marrow [172].

González-Trujano et al. [151] exposed mice to *S. divinorum* non-polar, medium polar, and polar extracts at doses of 10, 100, 1000, and 2000 mg/kg (i.p.). During the following 14 days, no macroscopic organ injury or weight loss were observed, although diarrhoea-type faeces were reported for mice receiving 1000 and 2000 mg/kg of the non-polar extract. Authors determined an LD_50_ of 1000 mg/kg for non-polar and medium polar *S. divinorum* extracts, and 1414 mg/kg for the polar extract [151].

Socała et al. [173] have recently evaluated in albino Swiss mice the possible influence of salvinorin A (0.1, 1, and 10 mg/kg, i.p.) on seizure susceptibility, observing that the hallucinogen had no significant effect on the thresholds for first myoclonic twitch, generalised clonus, and tonic forelimb extension, and on the current intensity necessary to induce hindlimb tonic extension and psychomotor seizures. Therefore, it was concluded that salvinorin A has no effects on seizure thresholds. Additionally, salvinorin A had no impact on muscle strength and motor coordination [173].

Martinho et al. [174] demonstrated dose- and time-dependent cytotoxic effects of *S. divinorum* and salvinorin A (0, 0.1, 1, 10, or 50 μM), evaluated through MTT (3-(4,5-dimethylthiazol-2-yl)-2,5-diphenyltetrazolium bromide) assays, in N27 dopaminergic neurons, COS-7 lung, Hek 293 embryonic kidney, HepG2 liver, and intestinal epithelium Caco-2 cells.

## 12. Abuse Potential, Dependence, and Tolerance

### 12.1. Effects in Humans

Abuse liability of *S. divinorum* appears to be lower when compared with that for other substances that are used recreationally [19]. This plant is sporadically consumed in recreational contexts, does not induce a compulsive/persistent administration behaviour, and there have been descriptions of consumers feeling aversive effects towards salvinorin A [4,82], which suggest low dependence liability. In fact, some users report a dislike of the effects induced by the drug, leading them to stop its consumption [53,144,145].

Little evidence of dependence on *S. divinorum* was disclosed in one anonymous web-based survey [15], but withdrawal symptoms (e.g., anxiety, irritability, uneasiness, nausea, and vomiting) and strong craving for *S. divinorum* were reported after discontinuation of chronic consumption of *Salvia*, with the individuals showing an exacerbation of social and relational problems, suggesting the development of a substance use disorder [164,175]. Additionally, individuals stated they required increasing amounts of *S. divinorum* to achieve the desired effects, this being suggestive of the development of tachyphylaxis [176].

### 12.2. Animal Testing

In animal models, behavioural conditioned place aversion was developed in mice towards salvinorin A (doses of 1.0 and 3.2 mg/kg, i.p.), reinforcing the non-addictive feature of this psychoactive substance [132]. When administered at low doses (from 0.1 to 80 μg/kg, i.m.), salvinorin A displayed abuse potential in zebrafish [128]. The same results were obtained after s.c. injection of low doses of salvinorin A (0.01–40 μg/kg) into Wistar rats [129], further supported by an increase in DA extracellular levels in the nucleus accumbens. Notwithstanding, at high doses (160 μg/kg), an aversive effect towards salvinorin A was observed [128,129]. Of note, the low doses used in these studies are similar to those commonly smoked by humans (3–7.5 μg/kg). Serra et al. [136] observed only small changes in extracellular levels of DA in the nucleus accumbens of male Lister hooded and Sprague Dawley rats, after administration of salvinorin A (5–40 μg/kg, s.c.). Recently, after i.p. administration of *S. divinorum* ethyl acetate extract to Wistar rats, no modifications of DA levels in the nucleus accumbens were observed [177]. Data from these in vivo studies suggest that *S. divinorum* and salvinorin A effects may depend on the animal model/strain, doses, and administration route.

## 13. Potential Therapeutic Benefits

### 13.1. Remediation and Prevention of Gastrointestinal Dysfunction

Intraperitoneal (6 mg/kg/day) and oral (10 mg/kg/day) administration of salvinorin A to two experimental mice models of colitis demonstrated therapeutic potential for the treatment of inflammatory bowel disease [156]. In a mouse model of LPS-induced endotoxemia, treatment with salvinorin A (3 mg/kg, i.p.) resulted in decreased intestinal barrier permeability, preventing its dysfunction, decreased small intestinal hypermotility, and normalisation of neurogenic ion transport [155]. Exposure of isolated mice smooth muscle strips to salvinorin A (3 mg/kg) led to reduced contractions of the colon, stomach, and ileum, and inhibited neurogenic ion transport; in Swiss albino mice, exposure to the drug (3 mg/kg) increased colonic expulsion time [108]. These actions of salvinorin A were reportedly mediated by KOP, CB1, and CB2 receptor activation [108].

A standardised extract from *S. divinorum* leaves (1–100 mg/kg) and isolated salvinorin A (0.01–10 mg/kg) were given i.p. to mice, demonstrating an absent or weak (only at the highest doses of salvinorin A) effect in the healthy control mice, while in a mouse model of chronic small intestine inflammation, there was a normalisation of the intestinal motility that occurred through a KOP-dependent mechanism [158]. These data corroborate the long-recognised potential of salvinorin A for the management of gastrointestinal illnesses (e.g., diarrhoea, inflammatory bowel disease), and its possible use for the development of new drug candidates that might be able to affect pathophysiological tissues without significant properties under physiological states [158].

### 13.2. Antidepressant/Pro-Depressant and Anxiolytic Properties

A 26-year-old woman, to whom *S. divinorum* leaves (0.5–0.75 g) were sublingually administered three times per week, reported a complete remission of depressive symptoms after 6 months, as measured by the Hamilton Depression Scale [178]. Antidepressant and anxiolytic properties were also shown in rats (0.001–1000 μg/kg, s.c.) [150]. However, other studies in animals have shown depressive-like symptoms (e.g., reduced motivation and locomotor activity) for high salvinorin A doses (0.125–2.0 mg/kg) [133,179], accompanied with decreased DA release in the nucleus accumbens. At such drug levels, a possible therapeutic benefit to control symptoms of mania can thus be raised [180], as well as other disorders featuring hyperfunction of the DA system [133].

### 13.3. Neuroprotection

It was demonstrated both in piglet (10 μg/g, i.v.) and C57BL/6J mouse models (0.5 mg/kg, i.p.) that salvinorin A could have therapeutic benefits for neonate brain protection [181,182,183]. The drug causes cerebral vasodilation, conserves artery autoregulation, reduces mortality rate, and improves several short-term neurological developmental outcomes, leading to a reduction in neonatal hypoxic–ischaemic-induced injury [181,182,183]. Su et al. [184] evaluated effects on the cerebral vasculature, following the administration of 10 nM and 1 μM of salvinorin A to newborn pigs. Salvinorin A dose-dependently dilated the pial artery, both under normal and vessel-constricted conditions induced by endothelin and hypocarbia, with this effect being blocked by a nitric oxide synthase (NOS) inhibitor, a K_ATP_ channel inhibitor, and by a KOP-selective antagonist, suggesting that the dilation effects induced by salvinorin A are mediated by NOS, K_ATP_ channels, and KOP activation and, therefore, the drug’s therapeutic potential in disorders where cerebral vascular dilation is necessary [184]. More recently, in a middle cerebral artery occlusion (MCAO) mouse model, it was demonstrated that specific KOP activation by salvinorin A (intranasal administration of 12.5, 25, and 50 μg/kg) led to a dose-dependent decrease in cerebral infarct volume, protection of the vascular integrity, and improvement of neurological development [185]. An increase in IL-10 levels, restoring them to basal values, was also observed, thus consistent with anti-inflammatory properties of salvinorin A. A decrease in caspase-3 activation was also reported, this being coincidental with the neuroprotective properties of the drug through the inhibition of neuron apoptosis, protecting them from transit into infarct [185].

Salvinorin A improved inflammation and oedema in a transient global cerebral ischaemia Sprague Dawley rat model, after i.v. administration of two doses (10 and 20 μg/kg) [186]. The drug was capable of preserving autoregulation of the cerebral pial artery, reducing cell apoptosis, which results in the protection of the brain against ischaemia injury, and improving motor function. These effects were suggested to occur via the phosphoinositide 3-kinase (PI3K)/protein kinase B (Akt)/cyclic guanosine monophosphate (cGMP) pathway, since increased expression of Akt phosphorylation and increased levels of cGMP were observed.

Salvinorin A (20 μg/kg, i.v.) protected cerebral vessels against injury induced by ischaemia/reperfusion in a MCAO Sprague Dawley rat model, and in an oxygen–glucose deprivation (OGD) human brain microvascular endothelial cell (hBMEC) model [187]. A reduction of cerebral oedema and improvement of BBB permeability were reported in the MCAO model, while in hBMECs, salvinorin A reduced damage and apoptosis. Furthermore, the drug protected neuronal mitochondria function after cerebral ischaemia, as verified through activation of adenosine monophosphate-activated protein kinase increased mitofusin-2 expression, reduction of oxidative stress, stabilisation of mitochondrial membrane potential, and preservation of morphology and functions of mitochondria in cerebrovascular endothelial cells [187].

Another clinical application of salvinorin A was recently proposed, involving the treatment of cerebral vasospasm and ischaemia as a result of subarachnoid haemorrhage (SAH) [188]. SAH Sprague Dawley rats exposed to salvinorin A (2 and 10 μg/kg, i.p.), had an increase in the diameter of the basilar artery, a decrease in the thickness of the basilar artery wall, and an improvement of the morphological changes in the serrated inner elastic membrane. It was suggested that salvinorin A could lead to a significant improvement in neurological function. Parallel to these beneficial effects, an increase in the concentration of the vasodilator nitric oxide and a decrease in the levels of the vasoconstrictor endothelin-1 have been observed. Salvinorin A also induced the downregulation of membrane water channel aquaporin-4 (AQP-4), thus possibly contributing to the amelioration of brain injury, as this protein is involved in the establishment of brain oedema after stroke [188]. In SAH animals exposed to 10 μg/kg i.p. salvinorin A, an inhibition of the lateral ventricle expansion and improvement of early brain injury were observed [189]. This therapeutic effect was supposedly accomplished via salvinorin A activation of the KOP and PI3K/Akt signalling pathway. Consequently, the levels of apoptosis-related proteins (FoxO1, Bim, Bax, and cleaved caspase-3) were decreased, as well as those related to inflammation (p-IKKα/β and NF-κβ) [189].

A potential therapeutic benefit for the treatment of diseases caused by hypoxia/ischaemia (e.g., behavioural dysfunction, mental retardation, learning difficulties) was associated with salvinorin A [183]. Xin et al. [190] explored the drug’s potential for protection and treatment of injury induced by brain ischaemia/reperfusion. Using a forebrain ischaemia/reperfusion injury Sprague Dawley rat model exposed to salvinorin A (0.01 μg/g, i.v.), a significant reduction of AQP-4 expression in the hippocampus, cortex, and striatum was observed. This results in decreased neuronal tumescence and attenuation of neuronal death, contributing to an improvement in brain oedema, the lessening of neuronal apoptosis, and decreased reperfusion-induced cerebral injury. Furthermore, the rats with forebrain ischaemia had a significant amelioration of motor and cognitive functions following salvinorin A treatment [190]. More recently, acute intranasal administration of salvinorin A (25 μg/kg) in a stroke rhesus monkey model highlighted its therapeutic potential for stroke rescue as the drug significantly decreased the brain infarct volume and improved neurological outcomes, neuroprotective effects that were maintained for 28 days after the procedure [191].

### 13.4. Anti-Inflammatory and Antinociceptive Properties

Aviello et al. [159] investigated anti-inflammatory properties of salvinorin A in mice. Treatment at the lower dose (0.5 mg/kg, i.p.) led to a significant reduction in LPS- and carrageenan-induced paw oedema, while the higher dose (2 mg/kg, i.p.) was effective in prevention of the second phase (i.e., inflammatory pain) of the nociceptive behaviour induced by formalin. Salvinorin A-moderated anti-inflammatory effects in vivo occurred via KOP and CB1 receptor-mediated pathways. As such, by targeting KOP and CB1 receptors, salvinorin A might be used as a new, interesting lead compound for the development of novel anti-inflammatory drugs [159].

A new therapeutic target for salvinorin A was recently identified, as the drug induced a dose-dependent inhibition of leukotriene (LT) biosynthesis in macrophages, which are potent mediators of inflammation [192], both in vitro (tested in activated rat peritoneal macrophages, at 0.01–1 μM) and in vivo (tested in zymosan-induced peritonitis mouse and carrageenan-induced pleurisy rat models, at 1–10 mg/kg, i.p.). Besides the inhibition of LT biosynthesis, salvinorin A also inhibited cell infiltration and myeloperoxidase (MPO) and vascular permeability in the peritoneal cavity of the peritonitis mouse model, as well as decreased exudate volume, inflammatory cells, and MPO activity in the pleurisy rat model [192]. The possibility of using salvinorin A for the treatment of LT-related diseases (e.g., asthma, allergic rhinitis, and cardiovascular diseases) was therefore suggested, highlighting potential benefits in the control of allergic inflammation [193]. In an air pouch mouse model, salvinorin A (10 mg/kg, i.p.) was able to inhibit pulmonary mast cell degranulation and LT and IL-13 production, culminating in reduced bronchial hyperreactivity [193].

Simón-Arceo et al. [177] evaluated neuropathic antinociceptive effects in a neuropathic pain Wistar rat model, following administration of an *S. divinorum* extract (30, 100, and 200 mg/kg, i.p.). *S. divinorum* demonstrated transient antihyperalgesic effects (at 100 and 200 mg/kg), and an increase in paw withdrawal latency (only at 100 mg/kg). These results corroborate the possibility of using salvinorin A as an alternative therapy for neuropathic pain. Mascarenhas et al. [194] showed that salvinorin A has significant analgesic properties at high doses (0.05–0.45 mg/kg) in infant 21-day-old rats through a reduction in formalin pain scores. Guida et al. [195] evaluated the effect of salvinorin A administration (0.5, 1, and 2 mg/kg, i.p.) on pain induced in Institute for Cancer Research (ICR) mice treated with formalin. An antiallodynic effect promoted by salvinorin A was observed, with this being mediated by KOP and CB1 receptors. Analgesic and anti-inflammatory properties of acute salvinorin A (1–2 mg/kg, i.p.) administration in mice were also recently demonstrated in a formalin assay [196], which included a reduction in mechanical and cold allodynia in the paclitaxel-induced neuropathic pain model.

### 13.5. Treatment of Drug Dependence

Locomotor, affective, and cognitive effects provoked by cocaine consumption may be modulated by KOP activation [148]. Along this line, salvinorin A was shown to act as a dose-dependent punisher of cocaine and remifentanil in rhesus monkeys, reducing self-administration of these drugs [197]. The therapeutic potential of salvinorin A for the treatment of drug dependence comes from the drug’s capacity to decrease dopaminergic activation and extracellular DA levels [197].

## 14. Conclusions and Future Perspectives

The use of psychoactive herbal drugs aimed at religious, mystical, or healing purposes has been part of human history for centuries. In spite of these natural compounds being described for years in ethnobotanical histories of specific cultures, they often arise in non-endemic settings as novel, alternative legal highs. Clinicians are oftentimes unaware of these emerging recreational substances, as well as of their biological effects, leading to erroneous diagnostics, as many are not detected in ordinary drug screening methods.

**Figure 6:**
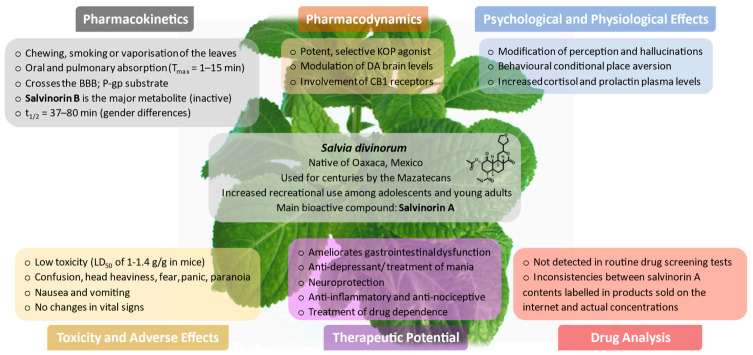
Toxicokinetic and toxicodynamic aspects of *Salvia divinorum* and salvinorin A. BBB: Blood–brain barrier; CB1: Cannabinoid receptor 1; DA: Dopamine; KOP: κ-Opioid receptor; P-gp: P-Glycoprotein.

*S. divinorum* is a psychotropic plant endemic to Oaxaca, Mexico, used for centuries by the Mazatecans for medicinal and divinatory purposes, and whose well-known bioactive compound is the neoclerodane diterpene salvinorin A. Intense and short-lived psychological and physiological effects are felt after its consumption, predominantly mediated by an efficacious and selective KOP agonistic activity. A summary of the main findings regarding the pharmacokinetics, pharmacodynamics, biological effects, and clinical and forensic relevance of *S. divinorum* and salvinorin A addressed in this article can be seen in Figure 6.

Due to the widespread legal availability and consequent increase in recreational misuse, both psychopharmacology and abuse liability of *S. divinorum* and salvinorin A need to be comprehensively evaluated and described [19]. It is noteworthy that the drug seems to induce tolerance without displaying abuse potential nor dependence. Toxicological effects derived from misuse are rare and mild, with the risk of confusion, lethargy, heaviness of head, tachycardia, and paranoia increasing either with dramatic dose escalation or with polydrug abuse of alcohol and other drugs.

Of concern, there is a lack of knowledge on the complete pharmacokinetic profile, metabolism, and long-term effects of *S. divinorum* and salvinorin A. Additionally, its exact and complex pharmacodynamic mechanisms, that lead to both desired pharmacological but also adverse side effects, need a better, holistic understanding that can only arise from the complete articulation between the cellular, behavioural, and pharmacokinetic profile [161]. For the treatment of pain, some neurological and neuropsychiatric disorders (e.g., Alzheimer’s, schizophrenia, bipolar disorder) and drug addiction have taken advantage of the use of the KOP receptor as a key target [8,198,199,200]. In this sense, there is growing interest in natural salvinorin A as a lead compound for the discovery of other more effective drug candidates [27,30,146], particularly because the drug seems to be deprived of moderate to high toxicity, displaying an interesting safety vs. efficacy profile.

A variety of preclinical studies suggest conceivable therapeutic applications for salvinorin A as an analgesic, anti-inflammatory, neuroprotective medicine, amongst other clinical applications. However, the relatively short duration of physiological/psychological effects in vivo has been also demonstrated, as well as the limitations of the administration route (oral route not viable), and the undesirable psychotropic activity that hampers its clinical acceptance.

Future directions to improve the safety and therapeutic potential of salvinorin A in humans might rely on drug molecular modification. As such, the scientific community has turned to the development of new, longer-lasting, orally available, structurally related synthetic/semi-synthetic analogues, with the same therapeutic properties (e.g., analgesic, anti-inflammatory, treatment of drug dependence, etc.), but lacking the unwanted psychotropic activity [92]. Molecular modifications of salvinorin A, rendering different analogues, include substitutions or changes of the C2 acetate group, C4 methyl ester group, and furan ring [201], that might feed the current investigational clinical pipeline. In this sense, a salvinorin analogue containing an *N*-methylacetamide moiety at the C-2 position [180] proved to be a new pharmacological tool with advantages regarding the duration of behavioural effects and oral availability. Another synthetic derivative, salvinorin B methoxymethyl ether, proved to be more potent than salvinorin A, inducing antinociception effects, and having antiaddiction properties towards cocaine consumption with a longer-lasting action [202,203]. The development of a salvinorin A aromatic analogue, salvindolin, was recently proposed for therapeutic purposes through modification of the C2-side chain with a 2-indolyl moiety [78]. This new molecule showed antinociceptive effects mediated by MOP and KOP receptors, and antidepressant-like properties mediated by MOP/5-HT_1A_ receptor activation, without the psychotropic side effects usually associated with salvinorin A. Another aromatic analogue of salvinorin A obtained through the integration of a benzoyl moiety at the C2-position, herkinorin, was previously reported as the first non-nitrogenous compound with greater affinity for the MOP receptor over the KOP receptor [78,204]. This compound, unlike other MOP agonists, did not induce MOP signalling through the β-arrestin-2 pathway nor promote receptor internalisation. Since the activation of the β-arrestin-2 pathway modulates several opioid-related adverse effects, such as tolerance, constipation, and respiratory depression, herkinorin can be ultimately used for the development of opiate analgesics with a better safety profile [205]. However, the administration of high herkinorin concentrations through systemic injections is difficult, as this compound has low stability and poor water solubility, limiting its therapeutic application [205]. Of note, the positive identification of herkinorin was reported in a non-targeted novel psychoactive substance screening applied to agitated patients in an emergency department [206], suggesting its recent use as a recreational drug of abuse.

## Figures and Tables

**Table 1:** Concentrations of salvinorin A and salvinorin B reported in different *S. divinorum* samples.

Sample	Salvinorin A (mg/g)	Salvinorin B (mg/g)	Reference
Leaves from private collections ^1^ and endemic populations of Oaxaca ^2^	0.89–3.70	-	[41]
Leaves from plants endemic to Sierra Mazatecan	7.6	4.20	[39]
Leaves from Hawaiian plants	7.8	10.4	[39]
Leaves and extracts purchased on the Internet (1–20×)	0.126–1.137	-	[40]
Dried leaves and concentrated extract products purchased on Japanese drug market (1×)	3.2–5.0	0.10–0.17	[22]
Ground young leaves of plants purchased from the Vancouver Seed Bank	0.9		[42]

^1^ Wasson and Hofmann clones and “Palatable” clones. ^2^ Cerro Rabón collection and Huatla de Jimenez collection. (-): Not mentioned.

**Table 2:** Detection and quantification of salvinorin A in biological matrices and *S. divinorum* plant material.

Constituent	Matrices	Sample Preparation and/or Methodology	Linearity (R^2^)	Intra-Day Assay		Inter-Day Assay		LOD (μg/mL)	LOQ (μg/mL)	Recovery (%)	Reference
				Precision (%CV)	Accuracy (%)	Precision (%CV)	Accuracy (%)				
Salvinorin A	Methanol extract of *S. divinorum*	icELISA	-	<3.19	-	2.78–9.65	-	0.0195	-	96.15–104.10	[94]
Salvinorin A	Plasma; urine; saliva; sweat	LLE; GC-MS	>0.99	<13.0	-	<15.0	-	0.05 for plasma, urine, saliva; 0.03 for sweat	0.015 for plasma, urine, saliva; 0.010 for sweat	77–94	[68]
Salvinorin A	Human plasma; rhesus monkey CSF	LC-MS/MS-ESI	-	<2.85 for plasma; <1.3 for CSF	<7.08 for plasma; <4.94 for CSF	<3.47 for plasma; <1.7 for CSF	<2.37 for plasma; <9.42 for CSF	-	0.00005 for plasma; 0.0000125 for CSF	93–114 for plasma	[98]
Salvinorin A	Urine	LC-MS-ESI	0.997	<7.5	<9.5	<8.5	<6.2%	0.0025	0.005	-	[99]
Salvinorin A	Hair	UHPLC-MS/MS	0.0997	<12.0	-	<7.0	-	0.00002 μg/mg	0.00005 μg/mg	76.6–97.4	[100]
Salvinorin A	Urine	MEPS; GC-MS/MS	>0.99	<11.0	<9.0	<8.0	<8.0	0.005	0.02	71–80	[96]
Salvinorin A	Human plasma	SPE; HPLC-APCI-MS	0.999	<11.2	96.4–104.0	<10.3	98.7–101.1	0.002	0.002	-	[89]
Salvinorin A	Pericardial fluid; vitreous humour; whole blood; plasma	SPE; GC-MS-EI	>0.99	<12.0	9.0	-	-	0.005	0.005	79.65–99.09	[97]
Salvinorin A	*S. divinorum* products sold throughout Mexico	HPLC	>0.999	<5.0	-	<2.0	-	0.44	1.34	-	[73]
Salvinorin A Salvinorin B Salvinorin C	*S. divinorum* products obtained online and in Portuguese smartshops	GC-MS	>0.99	3.6–8.6	-	6.6–14.9	-	1.25 μg/mg	2.5 μg/mg	-	[104]

CV: Coefficient of variation; CSF: Cerebrospinal fluid; EI: Electron ionisation; ESI: Positive electrospray ionisation; GC-MS: Gas chromatography–mass spectrometry; GC-MS/MS: Gas chromatography–tandem mass spectrometry; HPLC: High-performance liquid chromatography; HPLC-APCI-MS: High-performance liquid chromatography–atmospheric pressure chemical ionisation mass spectrometry; icELISA: Competitive enzyme-linked immunosorbent assay; LC-MS/MS: Liquid chromatography–tandem mass spectrometry; LOD: Limit of detection; LLE: Liquid–liquid extraction; LOQ: Limit of quantification; MEPS: Microextraction by packed sorbent; SPE: Solid-phase extraction; UHPLC-MS/MS: Ultra-high pressure liquid chromatography tandem mass spectrometry; (-): Not mentioned.

## Data Availability

Not applicable.
